# Edinburgh Royal Infirmary

**Published:** 1892-12-10

**Authors:** 


### The Institutional Workshop.

### WITHIN THE HOSPITALS.

### EDINBURGH ROYAL INFIRMARY.

(By Our Special Commissioner.)

It is an education to spend a few days at Edinburgh in the very pleasant occupation of studying the administration of the Royal Infirmary. There can be no doubt, that viewed from every aspect, and examined by the eye of experience, the Royal Infirmary, Edinburgh, at the present time is a hospital of which the British nation, as well as the citizens of Edinburgh, may be justly proud. There is nothing quite like it in any other part of the British Empire, and it has a prestige all its own. In every department the same spirit of thoroughness and *esprit de corps* makes itself felt. We take it that if the average pessimist could be made to understand a little of the details of hospital administration, and then be brought to Edinburgh, and caused to realise the work of each member of the staff of the Royal Infirmary, from the honorary physician and surgeon to the humblest worker in the wards, he would find it very difficult to keep up his pessimism. Everybody seems to feel that it is an honour to belong to such an institution, and that they must individually take care that nothing on their part shall be wanting to contribute to the success of the whole machine.

It is always instructive for the trained administrator to visit and carefully inspect great institutions. When the administration is as good as human devotion to duty can make it, then the pleasure and instruction are greater than words can adequately describe. We felt at Edinburgh, as we felt last year at the Johns Hopkins Hospital, Baltimore, that it was a great privilege to be called to work in either of these institutions. The effect upon the *morale* of the staff must be fruitful for good. Whether the workers' stay there be long or short, its influence is likely to affect them favourably throughout their whole life. The knowledge of this should make every thoughtful man or woman zealous of the good name and progressive development of our great medical institutions. Their influence for good, apart from the immediate object which they fulfil, is enormous when rightly understood. Inefficiency and failure to secure the best, for the want of a little enterprise or the expenditure of a few pounds, should be regarded as a defect which it is incumbent upon public opinion to remedy without delay. At least, and as a mere act of justice, we are bound to declare, that the excellent scientific work, and the efficiency of the administration at the Royal Infirmary, Edinburgh, must have a marked influence for good, not only upon those who labour, but also upon those who suffer within its walls. Edinburgh has good reason to be proud of this great institution.

#### Character and Cost of the Work.

It would be an act of supererogation to give a detailed description of the buildings of this great pavilion hospital in these columns to-day. Suffice it to say that the wards are admirable, and that the plan of providing separate small wards in juxtaposition to the larger ones, together with rooms for clinical and other purposes, is a good one. It was pleasant to notice that every sister was not only proud of her work, but that, with the earnest and continuous co-operation of the physician or surgeon in charge, each sister aims to make her own ward the brightest and most attractive of the whole establishment. This friendly rivalry produces on the whole a result which is surprisingly satisfactory. We feel that hospital managers might usefully take a leaf out of the book of the asylum superintendents, and that when the time comes round to have the wards redecorated, care should be taken that the decorations in each ward should differ from those in other wards, and that they should harmonise and be made as attractive and pleasant as possible. These effects can be obtained without much, if any, additional outlay, the one thing necessary is the presence of someone who has enough knowledge of decoration and sufficient taste to secure the best effect possible. Rightly understood, there can be no doubt that the tasteful decoration of a hospital, which should always present the brightest and most cheerful aspect it is possible to attain, cannot fail to have the happiest results upon the patients, whose recovery is thereby hastened, and upon the visitor, who experiences so sincere a pleasure that he cannot resist the temptation of asking to be allowed to contribute—and to contribute liberally—to the maintenance of an institution which exhibits at a glance the maximum of care and the presence of an earnest desire to make the house of suffering as attractive and pleasant as possible. Of course, although the wards at Edinburgh are bright and pleasant to look upon, although they contain many flowers and pictures, still much more is capable of being done in the way of effective decoration. Such a development will no doubt have its effect upon the appearance of the whole of the interior of the Royal Edinburgh Infirmary in due time.

Turning now to the cost of the work, we find from the last report that there is a tendency in Edinburgh as elsewhere for the cost per bed to increase. The attention now paid to cleaning, decoration, special appliances used for heating and lighting, replacing the old by new and better furniture, and improved nursing, all tends to force the expenditure upwards. Six hundred and forty-eight beds were constantly occupied, and the average period of residence was from 23 days in the Lock Hospital to 27 days in the ordinary medical wards, being an average of 26·9 days per patient. Altogether 8,769 in-patients were under treatment, of which 3,878 were medical and 4,281 were surgical cases. The cost of each occupied bed in 1891 was £62 10s., compared with £58 16s. for the previous year. Despite this increase the cost of food per occupied bed has remained stationary at £20 4s. 5d. per bed. The invested property of the infirmary under all heads amounts at the present time to £296,158 in round numbers.

#### The New Nursing Home.

We have been led to refer above to hospital decoration by the fact that the new Nurses' Home, now nearly complete, which will be opened shortly, has been erected upon the right principle, and promises to be the most pleasant building which has yet been erected as a residence for nurses. For example, nearly all the rooms are made to differ a little in shape, all have been decorated in carefully varied colours, the roof of the corridors on the first floor has been so constructed as to give a most pleasant effect to the eye, and the floors will be covered, we understand, with Brussels carpet. Now the public may laugh, but it is perfectly accurate to say that Brussels carpet, rightly used, may be made an education in itself. It gives a sense of pleasure to those who are constantly moving about in a building of this character; it tends to make the whole establishment quiet, and free from unnecessary noise, and, by exciting a feeling of satisfaction amongst those who reside in the home, cannot fail to raise the *morale* of the whole staff. Realising all this, and having by practical experience learned its importance, we do most heartily thank those who are responsible for the plans, the interior decoration, and the furniture of the Nurses' Home of the Royal Edinburgh Infirmary for the important service they have rendered to nursing in this country. No praise can be too high for such devoted vigilance as has been shown by Surgeon-General Fasson, the late superintendent, whose recent death has caused a feeling of sorrow throughout all the workers in the Infirmary, and Miss Spencer, the very able lady superintendent. There is, we know, a feeling among better educated ladies who become nurses that there is only one place after all for which they should make, and that place is London. The inspection we have made of the larger hospitals on the eastern side of England and in the chief towns of Scotland convinces us, however, that the time is not far distant when this feeling will be largely discounted by the fact that the accommodation provided, and the character of the work afforded, to nurses in the larger centres of population all over the country, are now so good, that anyone of these ladies ought to be proud to have charge of any of their great institutions.

### Nurses and the Public Institutions.

Much inconvenience has been caused for years to the nurses of all grades at the Royal Edinburgh Infirmary. They have had to sleep in large numbers in the roofs of the pavilions, being scattered all over the establishment, and their comfort and happiness have consequently been sorely interfered with. Still the nurses had confidence that those who were in authority were doing all they could to relieve this discomfort, and it is highly creditable to the institution that there has never been a single word of complaint of any kind or description. We commend this circumstance to the notice of those who are so fond of stirring up discontent. It is quite clear that in a large establishment, especially when the buildings are old, the difficulties of meeting the just requirements of everybody become at times almost insurmountable. Still, if there is one thing connected with the administration of the medical institutions of this country to-day about which there can be no reasonale doubt, it is, that nearly everywhere the governing body take the warmest interest in the staff, and that on the whole they are most desirous of doing everything that is reasonable to secure their comfort and to promote the happiness of every individual worker. If nurses, for example, could only realise their best interests, they would certainly follow the course adopted by the nurses at Edinburgh of patient endurance and trust, because it is the wisest for them to follow on every ground. A nurse after she has left the hospital where she was trained is still an object of interest to the authorities, because the reputation of the institution is largely wrapped up in her future work and conduct. This truth, we hope that the public may speedily come to see and to act upon when they employ nurses in their private houses. All they have to do when they require a nurse, is to communicate with the matron of the nearest hospital, or to obtain one from a recognised institution, and to ask her to produce a certificate of training. Then, if anything goes wrong, they can trust the matron at the hospital to secure a thorough investigation and a just decision on the points at issue. Every nurse must know that she is not likely to secure from strangers anything like the measure of justice she will get from her own hospital authorities, because their interests are her interests, and for all practical purposes both are identical. Any person or Association which labours to disturb this feeling, or to prevent the course indicated from being universally adopted, should be discountenanced by the public, and avoided by every nurse of experience and common sense.

### Special Features.

In the course of our visit we went through the laundry, which is small for the immense amount of work that has to be done. Despite this, everything was in order, and the colour of the linen and all other articles which had been washed was remarkable. We have never seen anywhere more excellent work, and can only wish that it was possible to get washing done in London as it is done at the Edinburgh Infirmary, and, for that matter, at all the Scotch hospitals which we visited, where they have a laundry of their own. The system at Edinburgh is to place the laundry in charge of a superintendent, and Miss Spencer has certainly been most fortunate in her selection of a lady for this post. Her intelligence, her full mastery of detail, the excellence of the results she secures, and the quiet and rapid way in which the work proceeds are beyond praise. The whole of the kitchen, too, is under the management of a woman cook, a fine specimen of her class, who seemed to enjoy the work although it is most laborious at times. The stores, too, were excellent, and the arrangements for their issue, and the care which is evidently taken in their selection were most satisfactory. Everywhere, as we have said, *esprit* reigns supreme, and so the character of the work is uniformly good. We think the managers would be well advised were they to deal once and for all with the out-patient department. This is a blot at the present time on this great hospital at Edinburgh. It is altogether wrong to allow out-patients to hang about the wards until they can be seen by the medical staff. Such a system would not be tolerated elsewhere, and it is very surprising to find it remains at Edinburgh. The dangers of infection and the other drawbacks attending this practice must be patent to all. We mention it because we feel that the time has arrived when the out-patient question should be faced and settled upon right lines without delay.

